# Inequalities in health and health service utilisation among reproductive age women in St. Petersburg, Russia: a cross-sectional study

**DOI:** 10.1186/1472-6963-10-307

**Published:** 2010-11-11

**Authors:** Tatiana Dubikaytis, Meri Larivaara, Olga Kuznetsova, Elina Hemminki

**Affiliations:** 1 St. Petersburg Medical Academy of Postgraduate Studies, 193015 Kirochnaja ul. 41, St. Petersburg, Russia; 2 National Institute for Health and Welfare (THL), P.O. Box 30, 00271 Helsinki, Finland

## Abstract

**Background:**

Russian society has faced dramatic changes in terms of social stratification since the collapse of the Soviet Union. During this time, extensive reforms have taken place in the organisation of health services, including the development of the private sector. Previous studies in Russia have shown a wide gap in mortality between socioeconomic groups. There are just a few studies on health service utilisation in post-Soviet Russia and data on inequality of health service use are limited. The aim of the present study was to analyse health (self-rated health and self-reported chronic diseases) and health care utilisation patterns by socioeconomic status (SES) among reproductive age women in St. Petersburg.

**Methods:**

The questionnaire survey was conducted in 2004 (n = 1147), with a response rate of 67%. Education and income were used as dimensions of SES. The association between SES and health and use of health services was assessed by logistic regression, adjusting for age.

**Results:**

As expected low SES was associated with poor self-rated health (education: OR = 1.48; personal income: OR = 1.42: family income: OR = 2.31). University education was associated with use of a wider range of outpatient medical services and increased use of the following examinations: Pap smear (age-adjusted OR = 2.06), gynaecological examinations (age-adjusted OR = 1.62) and mammography among older (more than 40 years) women (age-adjusted OR = 1.98). Personal income had similar correlations, but family income was related only to the use of mammography among older women.

**Conclusions:**

Our study suggests a considerable inequality in health and utilisation of preventive health service among reproductive age women. Therefore, further studies are needed to identify barriers to health promotion resources.

## Background

Russian society has faced dramatic changes in terms of social stratification since the collapse of the Soviet Union. During this time, extensive reforms have taken place in the organisation of health services, including the development of the private sector [1]. Yet little is known about social differences in health and health service utilisation in Russia today.

Previous studies in Russia have shown a wide gap in mortality between socioeconomic groups, and the differences are greatest among young and middle age people with the largest relative difference in the age group 20-39 years [2]. However, mortality rate does not reflect the burden of disease attributable to chronic disabling disorders that are not life-threatening. Thus, more refined measures are needed in order to compare health differences between different social groups.

Self-rated health has been shown to be a valid and reliable measure of overall health status in other countries [3,4]. Previous studies of self-rated health in post-Soviet Russia have revealed that better self-rated health consistently correlates with higher household income and, in recent studies, with higher education as well. Some of the previous studies have been conducted in urban parts of Russia (Moscow [5], Taganrog [6,7]), while others cover the whole country [8,9].

In general, the variation in self-rated health by socioeconomic status can be explained by differing health behaviour, environment, genetic predisposition, and health service utilisation [10]. Previous studies have not, however, analysed differences in self-rated health together with utilisation of health services in Russia.

The current Russian health service system is based on mandatory health insurance that should guarantee universal access to public sector health services without user charges at the point of delivery [11-14]. In practice, patients have to contribute both semi-formal user charges and informal payments. The public sector primary health care in urban areas, such as St. Petersburg, is based on a network of neighbourhood clinics (polyclinics) that are responsible for the population in their catchment area. In addition, a network of specialist polyclinics, such as women's clinics, provides specialist services without the need for prior referral from the neighbourhood clinic. More specialised services are provided by hospital in- and out-patient units. Furthermore, a number of private clinics also provide health services in St. Petersburg today, and gynaecology is one of the specialties where private services have developed most rapidly [15].

Some clinics (public and private) have financial support from voluntary health insurance companies. A small number of employers of the richer companies tend to purchase voluntary health insurance for their staff to cover extra services provided by the clinics that have an agreement with private health insurance companies [16]. Not many people in Russia have voluntary health insurance (VHI) policies. Only 3% of those surveyed are holders of VHI policies [17].

There is very little data on how well the mandatory health insurance system has been able to maintain equal access to health services. A study comparing eight countries that were previously part of the Soviet Union revealed that in Russia, people with lower education refrain from seeking health services more often than those with higher education. Use of health services was markedly lower among those with fewer household assets, suggesting that equal access to health services has been compromised [18].

In this article, we studied inequalities in health and health service utilisation among reproductive age women in St. Petersburg, which is the second largest city in Russia. We examined whether self-rated health, self-assessed quality of life, self-reported chronic disease, and use of health services are associated with education and income level. One of our aims was to study whether the current health service system is capable of guaranteeing equal access to health services.

## Methods

The data of a representative survey of women aged 18-44 in St. Petersburg were used to analyse socioeconomic differences in self-rated health and utilisation of health services.

The survey was conducted in 2003-2004 in the catchment area of three women's clinics in two districts of the city, namely Krasnogvardeysky and Primorsky. The Krasnogvardejsky district consists of different areas with diversity in socioeconomic status. District residents are mainly employed in industry; the place is not very attractive because of higher environmental pollution and less convenient public transportation.

The industrial development of the Primorsky district was less intense during the Soviet period and this district is more attractive for those with higher income because of newer buildings.

The potential respondents were randomly selected. For the survey we did not have any formal sample size calculation. Based on feasibility 2.8% from a total of 90 532 reproductive age women were invited to participate. The original target sample size was 2501 women. The sample was drawn from a database maintained by the District Authority Police department.

The survey was approved by the Ethical Committee of the St Petersburg Medical Academy for Postgraduate studies. For a detailed description see earlier reports [19,20]. The survey questionnaire was anonymous and confidentiality was guaranteed [19,20].

A total of 782 women (31%) were excluded from the sample for the following reason: women were not reached due to a difference in their official address and their real living address or due to not reaching the person because of living abroad or elsewhere in Russia. The final sample consists of 1719 women, and the response rate was 66.7% (n = 1147). The recruitment procedure started with an invitation letter that informed about the purpose of the study and invited women to come to clinic. The invitation letter was followed by a phone call. During the phone call the location of the participant was verified, the willingness of the potential respondent to participate was confirmed and the participation method was agreed (clinic visit or completing the questionnaire at home).

The questionnaires were self-administered, consisting of multiple-choice questions. They contained several sections including background information, pregnancies and children, use of health services related to pregnancies and deliveries, values of childbearing, health and health behaviour, and use of health services [19,20].

### Measures

#### Independent variables

We used three different indicators of socioeconomic status (SES): education, personal income, and family income.

Education was divided into three categories: 1) school or college, 2) some university study, and 3) completion of university degree. This division was based on our awareness that people with a university education are more successful in building social networks and potentially could be more effective in looking for advanced medical care in comparison to those without a university degree. In our sample we had nobody without school education; therefore the lowest education category was defined as "school or college". Those who had started their university education, but did not complete it were considered as of the middle education group.

Personal income was classified into three categories based around the minimum living wage (MLW: 70 euro/month/person) in Russia at the time of the survey (low, signifying less than twice the minimum; middle, twice to less than four times the minimum; high, at least four times the minimum) [21].

The total family income was divided by the consumption units. Consumption units are calculated in the following way: the first adult = 1, the other family members = 0.8. After that family income was categorized in the same manner as personal income.

#### Dependent variables

Various indicators of health and utilisation of health services were used as dependent variables. Self-perceived health was assessed by question "How satisfied are you with your health?" Those who indicated being "very dissatisfied" or "dissatisfied" with their health were categorised as having poor self-rated health. Those who indicated being "neither satisfied nor dissatisfied", "satisfied" and "very satisfied" were used as the comparison group.

Chronic disease was assessed with the question "Do you have any permanent or chronic illness or any defect, trouble or injury that reduces your working capacity or functional ability?" Those who answered positively were categorised as having a chronic condition and those who indicated no chronic condition were categorized into the comparison group.

Quality of life was assessed with the question "How would you rate your quality of life?", with five possible answers were offered: excellent, good, average, poor and very poor. For the logistic regression analysis of the association between poor quality of life and SES we used the following dichotomisation: Quality of life was considered as poor if a respondent rated her quality of life as "poor" or "very poor" and those who answered "average", "good" and "excellent" were classified into the comparison group.

Information about the utilisation of out-patient services was collected through the question "Have you because of your own illness (or pregnancy or delivery), seen a physician during the past 12 months?" Those who answered "yes" were asked to indicate how many times they had seen a doctor along with the type of service providers: public sector polyclinic, a physician at an occupational health care centre, private clinic, seen a physician somewhere else. Those who indicated at least one visit were considered as an outpatient health care user. Based on this question four variables were created: public sector (polyclinic) utilisation, occupational health care utilisation, private clinic utilisation, and additional outpatient health centre utilisation. Variables were dichotomized in the following way: at least one visit = 1 and no visit = 0.

Hospital admission was probed with the question "Have you during the past 12 months been an inpatient in a hospital ward because of your own illness (or pregnancy or delivery)?" Those women who answered "yes" were categorized as having a hospital admission and those who answered otherwise were classified into the comparison group.

Utilisation of medical care during pregnancy has been assessed with the following question "Which health care provider have you visited during your last/current pregnancy?" with clarification of the type of medical centre: women's clinic, public health centre/aid station, private health centre, and some other centres. Based on this question four variables were created: women clinic utilisation, policlinics utilisation, private centre utilisation and other centre utilisation. Those who selected the specific type of medical centre were considered as a user of that kind of service and those who did not select the particular health care provider were classified into the comparison group. Only those who had their delivery in the 10 years previous to the study were included in the analysis.

Information about participation in preventive health examinations was obtained by the following question: "Have you had the following examinations during the past 5 years?: mammography, palpation of breast, ultrasonic examination of the breasts, PAP smear, a gynaecological examination". Based on this question four variables were created: mammography, ultrasound examination, PAP smear test, gynaecology examination. Those who selected the specific option were considered as a consumer of the particular examination and those who did not were classified into the comparison group.

The data analysis was performed with SPSS v. 13.0. Frequency analysis was used for the distribution of dependent characteristics by SES. Age-adjusted ORs and 95% CIs were calculated for self-rated health and health service utilisation by using logistic regression analysis, as presented in the tables.

Independent variables were entered simultaneously with age into the model equation in order to make an adjustment for age. Adjustment has also been done for socioeconomic covariates in order to analyse the independent influence of education and income on Pap smear, gynaecology examination and mammography.

The fitness of the logistic regression models were assessed with the Hosmer and Lemeshow test. Only those logistic regression models which had shown acceptable non-significant (p-value > 0.05) results were used for interpretation.

Pearson correlation between independent variables revealed no strong correlation between age, education level, personal and family income, suggesting no multicollinearity between independent variables. Collinearity diagnostics were done with the variance inflation factor (VIF) test; VIF was not higher than 1 and the tolerance statistic was more than 0,1.

## Results

The distribution of socioeconomic characteristics by age group is shown in Table S1 (see additional file 1). One-third (34%) of the study sample had a university degree. More than one third of women had a personal income less than 2 × MLW. The distribution of family income revealed that many women (41%) under 25 years of age did not know their family income. Among women aged 25-34, one-third and in the oldest group (aged 35+), 18% had no information about their family income. Only 7% of women reported a high family income.

The analysis revealed a significant association between socioeconomic status and health indicators (Table S2, see additional file 2). University education has a negative association with poor self-rated health, 35.7% of women with the highest education reported poor self-rated health in comparison to 45.5% of those with the lowest education. Women with a university degree were also less likely to assess their quality of life as poor: 10.6% vs. 5.6% among women with the lowest and the highest educational level, respectively. After adjusting for age the correlation between education and self-rated health was significant: women with low education were more likely to report that they had poor health status in comparison to those with a university degree (OR = 1.48, p = 0.011), in addition they were more likely to report that they have poor quality of life (OR = 2.02, p = 0.013). However, no association between level of education and chronic disease was found.

A borderline positive association between low personal income and poor self-rated health as well as chronic disease prevalence was revealed with logistic regression analysis (Table S2, see additional file 2). However, there was a strong positive relationship between personal income and self-assessed quality of life. Women with a low personal income were almost four times more likely to report a poor quality of life than those with high income (age-adjusted OR = 3.92, p = 0.005).

Family income was the strongest determinant of self-rated health. Half of women with low family income had rated their health as poor, in comparison with only a quarter of those with high family income (age-adjusted OR = 2.31, p = 0.003). Almost as strong an association was found in the prevalence of chronic conditions: women with low family income were more likely to report chronic conditions (age-adjusted OR = 2.00, p = 0.013). A strong relationship was also found between self-perceived quality of life and family income. As many as 14.1% of women with a low family income reported a poor quality of life, in comparison with only 1.2% of those with a high family income, (age-adjusted OR = 13.23, p = 0.012).

As for utilisation of health services, 58.8% of women had visited a physician during the past year independently of SES. The type of service provider varied significantly by socioeconomic status (Table S3, see additional file 3). Differences between educational groups were not discovered in the utilisation of policlinic, but university education was correlated with the use of additional outpatient services. 16.6% of respondents with a university degree had used private and 6.6% occupational health services, while, for comparison, only 5.6% and 2.7% of women with the lowest education had used private and occupational health services, respectively. After adjusting for age the correlation between education and outpatient service use was significant: women with a university degree were more likely to report private health care use (OR = 3.37, p < 0.001), and more likely to report occupational health care use (OR = 2.60, p = 0.016) in comparison to the respondents with low education. On the other hand, women with a university degree were less likely to have been hospitalised, with borderline significance (OR = 0.69, p = 0.078).

High personal income was strongly associated with the use of private services (age-adjusted OR = 3.56, p < 0.001), and middle income with occupational services (age-adjusted OR = 1.83, although with a borderline significance, p = 0.077); 23.4% of women with high income had visited a physician at a private clinic and 6.2% of women with middle income had used occupational health services. The association between personal income and hospitalisation was not found to be significant.

Family income had a specific influence on the utilisation of health services. Women with middle and high family income were significantly more likely to use private services (age-adjusted OR = 1.81, p = 0.048 and 2.81, p = 0.004; respectively) in comparison to the poorest respondents. Those who did not know their family income were also more likely to use private services, but this association was not statistically significant. However, those who did not know their family income were significantly less active in seeking public health services.

Table S4 (see additional file 4) describes health service utilisation during pregnancy. As in the case of overall utilisation, women with a university degree were more likely to visit private clinics (age-adjusted OR = 5.90, p = 0.007) and other centres (age-adjusted OR = 2.41, p = 0.051). In St. Petersburg, "other centres" most likely refers to highly specialised centres that are connected to teaching hospitals, medical academies and scientific research institutes.

Income was not significantly associated with health service utilisation during pregnancy.

Participation in preventive health examinations also varied significantly across socioeconomic groups (Table S5, see additional file 5). University education had a significant positive correlation with Pap smear and gynaecological examination (age-adjusted OR = 2.06, p < 0.001 and 1.62, p = 0.002; respectively) and high personal income had a significant positive correlation with mammography (age-adjusted OR = 3.32, p = 0.003), Pap smear (age-adjusted OR = 1.72, p = 0.004), and ultrasound examination (age-adjusted OR = 2.09, p = 0.005), while high family income correlated only with mammography for women over 40 years of age (age-adjusted OR = 4.34, p = 0.012). Women who did not know their family income were less likely to have undergone gynaecological examination.

The results of the multivariate analysis with mutual adjustment for all SES covariates (not given in the tables) revealed a significant association between university education and utilisation of medical examinations. Women with a university education were more likely to obtain a Pap smear test and gynaecological examination (OR = 1.58, CI = 1.09-2.30 and OR = 1.55, CI = 1.09-2.21, respectively). However, only personal income had an independent influence on mammography, (OR = 3.43, CI = 1.10-10.68) among those older than 40 years. Family income had no independent influence on health examinations.

## Discussion

The results reveal inequality in self-rated health, self-reported chronic disease, self-assessed quality of life and utilisation of health services across socioeconomic groups in reproductive-age women of St. Petersburg. The analysis suggests that socioeconomic determinants of health in comparison to socioeconomic determinants of health service utilisation are not the same. High personal income and university education, but not family income were associated with the use of additional health services and higher rates of participation in preventive health examinations. However, high family income was associated with better self-rated health, absence of chronic disease, and better self-perceived quality of life.

Some study limitations should be taken into consideration. First of all the cross-sectional design does not allow for any conclusions about causality in regard to health. It is possible that health-related social mobility may contribute to a positive association between health and higher income and education. However, that kind of reverse effect is not likely for socioeconomic differences in health care utilisation; for example, primary prevention measures such as cancer screening are unlikely to affect a person's socioeconomic status.

Reporting bias is another potential problem with a cross-sectional study. Women with low income may be more likely to report poor health. In this case the association between good self-rated health and high income may be overestimated. However, according to studies from other countries, self-rated health is a reliable measure of health status [3,4].

It is also possible that women with higher education are more likely to recall and report the exact names of tests they had undergone, e.g. Pap smear. However, given that women with lower education had also reported a lower frequency of gynaecology examination one can assume that the correlation between high education and Pap smear is not biased.

Selection bias may contribute to some data misinterpretation. If women with higher income did not participate in the study, the influence of society stratification may be underestimated. This study limitation is difficult to overcome given the lack of registers on education and income in Russia and the widespread underreporting of income.

The generalisability of the results requires a note, as well. St. Petersburg is a rather privileged and wealthy part of Russia and does not provide a typical example of the whole country. The population has a higher than average education, for example. In our study, the prevalence of a university degree is 34% and corresponds with official statistics [22]. Furthermore, the income differences are likely to be wider within St. Petersburg than within rural and semi-urban areas of Russia. However, it is likely that similar tendencies of inequality are to be found in other regions of Russia as well.

Our findings on the correlation between good self-rated health and higher education and income are consistent with the results of previous studies conducted in Russia [5-9]. However, in our study women reported poor health less often. In part this may be explained by the fact that our study population is younger.

A lack of association between self-reported chronic illness and education may have resulted from information bias; those with lower education may have been less aware of their illnesses. This view is supported by earlier findings that women with higher education are more likely to seek medical help at an earlier stage of the disease and use a variety of opportunities to control their health status more actively [23]. Therefore, they are more likely to discover the disease and get medical care at an earlier stage to preserve their health. On the whole, our results support the earlier view that those with higher education and income feel healthier and are more satisfied with their quality of life.

The results on utilisation of public health care services reveal hardly any differences between different socioeconomic groups. However, those with a university education visit a variety of service providers in addition to public sector services and those with a higher income visit the private sector more often. During the first two decades of post-Soviet Russia, both upward and downward social mobility were rapid and common. High income and high education do not necessarily go hand in hand in Russia, as they commonly do in Western Europe and the United States. Many highly educated occupational groups have been impoverished, while new business opportunities have paved the way to prosperity for those whose education may be lower.

Earlier studies from St. Petersburg have revealed how patients, as a result of being dissatisfied with public sector services, use a variety of strategies in order to gain access to better quality treatment and to less expensive or free-of-charge treatments [23-27]. These strategies tend to be patterned according to a person's socioeconomic status in such a way that those with higher education use their personal networks in order to access services, while those with lower education and relatively good financial resources prefer paid services. The socioeconomic patterning is likely to reflect the more extensive networks of those with higher education [25]. Against this background, it seems likely that the larger variety of health services used by women with a university education in our study is related to their personal networks, which enable them to access additional services regardless of their financial position.

High personal, and middle and high family income were related to more common use of private health services, but not with other additional services. Private services sometimes involve considerably higher user charges, but nevertheless they can be accessed easily if the user has the financial assets. Private services tend to be provided in more comfortable settings and they usually guarantee a higher level of privacy [15]. At the same time, distrust towards and dissatisfaction with public sector services is widespread [23-26]. Thus, it is not surprising that those who can afford it seek additional care from the private sector.

As for the utilisation of preventive health services, we found that, independently of personal and family income, women with a university degree were more likely to obtain a Pap smear and gynaecological examination, although in all education groups the prevalence of Pap smear and gynaecological examination was lower than recommended. Both examinations are essential and widely accepted health promotion measures for reproductive-age women. For comparison, a US study reported that 91.4% of reproductive-age women (18-40 years old) had been for a Pap smear, while in our sample the proportion was only 36.3% and 47% for those with a university education [28]. Our results suggest that the higher the woman's education, the more aware she is of the importance of preventive examinations. Financial barriers are unlikely to explain the whole story behind the underutilisation of the Pap smear and gynaecological examination, as the fee for the PAP smear is very small (less than 3 euro in 2004) and the latter can be obtained free-of-charge at public sector women's clinics. However, screening for oncology conditions can lead to the discovery of the disease, resulting in further treatment, which could act as a psychological as well as a strong financial barrier to the Pap smear among those worse-off, even though the Pap smear itself is cheap.

Women with higher personal income had a mammography more often, irrespective of their educational status and family income. This suggests that high personal income enabled women to have the diagnostic procedure done.

Thus, our study has shown underutilisation of medical services among women with low education and/or low income, despite universal health insurance coverage. In our survey we did not study the reasons for underutilisation. According to the international literature there is a variety of non-financial barriers to medical care consumption, though some of those, for example, distance between place of residence and medical centres, are unlikely to affect medical care utilisation because of the available transportation in St. Petersburg and the relatively close location of medical centres. However, potential non-financial barriers that deserve further clarification and future study include health beliefs, women's knowledge, lack of time for participation in preventive health examinations, an underestimation of the value of some health promotion measures, language limitations and provider bias.

## Conclusions

This is the first Russian study to show utilisation of preventive health services by socioeconomic status among reproductive age women. It indicates considerable inequalities in health and utilisation of health services. The results are especially evident in the utilisation of ambulatory health services, but not in hospital admissions. They suggest that mandatory health insurance is not working perfectly and that access barriers exist at least for health promotion measures.

In this study we analysed only a few diagnostic procedures as a proxy for the utilisation patterns of preventive medical care in general. Therefore, further studies are needed to identify barriers to health promotion resources.

## Competing interests

The authors declare that they have no competing interests.

## Authors' contributions

All authors jointly participated in development of study design. **TD **had participated in statistical data analysis and drafted the manuscript. **ML **had been involved in data interpretation and advanced the manuscript development. **OK **had been involved in revising the manuscript critically. **EH **helped in the manuscript improvement and had given final approval. All authors read and approved the final manuscript.

## Pre-publication history

The pre-publication history for this paper can be accessed here:

http://www.biomedcentral.com/1472-6963/10/307/prepub

## Supplementary Material

Additional file 1 **Table S1 "Distribution of socioeconomic status characteristics by age" is included into the file**. Click here for file

Additional file 2 **Table S2 "Prevalence (%) and age - adjusted OR of health indicators by education and income" is included into the file**. Click here for file

Additional file 3 **Table S3 "Prevalence and age - adjusted OR (95% CI) for use of different health care providers by SES" is included into the file**. Click here for file

Additional file 4 **Table S4 "Prevalence and age-adjusted OR for different types of health practices use during pregnancy by SES" is included into the file**. Click here for file

Additional file 5 **Table S5 "Prevalence and age-adjusted OR for preventive health care examinations by education and income" is included into the file**. Click here for file
