# Supplementary material for: Inequalities in health and health service utilisation among reproductive age women in St. Petersburg, Russia: a cross-sectional study
Source: BMC Health Serv Res. 2010 Nov 11;10:307. doi: 10.1186/1472-6963-10-307 (PMC2992514; doi:10.1186/1472-6963-10-307)
Supplement: Additional file 1 — Table S1 "Distribution of socioeconomic status characteristics by age" is included into the file. [file 1472-6963-10-307-S1.RTF]

Table 1. Distribution of socioeconomic status characteristics by age.

Socioeconomic characteristics	18–24	25–34	35–44	Totala	
	(n=304)	(n=349)	(n=492)	(n=1147)	
Education (%)	
School or college 	22.0	31.5	32.9	29.6	
Some university studies	55.9	26.6	29.7	35.7	
Completion of university degree	21.7	41.3	37.0	34.2	
Missing	0.3	0.6	0.4	0.6	
Personal income status (%)	
Low income (0–199%)	45.4	33.5	37.4	38.3	
Middle income (200–399%)	28.6	32.4	34.8	32.3	
High income >=400%	7.2	19.2	15.7	14.6	
Missing	18.8	14.9	12.2	14.8	
Family income status (%)	
Low income (0–199%)	13.2	21.8	33.9	24.7	
Middle income (200–399%)	16.4	19.2	23.6	20.3	
High income >=400%	7.6	9.7	5.5	7.4	
Woman does not know	41.1	32.1	17.5	28.2	
Missing	21.7	17.2	19.5	19.4	
aTotal includes 2 women who did not give information on age
