# Supplementary material for: Inequalities in health and health service utilisation among reproductive age women in St. Petersburg, Russia: a cross-sectional study
Source: BMC Health Serv Res. 2010 Nov 11;10:307. doi: 10.1186/1472-6963-10-307 (PMC2992514; doi:10.1186/1472-6963-10-307)
Supplement: Additional file 2 — Table S2 "Prevalence (%) and age - adjusted OR of health indicators by education and income" is included into the file. [file 1472-6963-10-307-S2.RTF]

Table 2. Prevalence (%) and age - adjusted OR of health indicators by education and income.


Socioeconomic characteristics	Poor self-rated health	Poor quality of life	Chronic disease	
	%	OR (95% CI)	p-value	%	OR (95% CI)	p-value	%	OR (95% CI)	p-value	
Education	
School or college (n=339)	44.5	1.48 (1.09–2.00)	0.011	10.6	2.02 (1.16–3.51)	0.013	35.4	0.97 (0.71–1.32)	0.825	
Some university studies (n=409)	37.2	1.24 (0.92–1.67)	0.158	7.6	1.52 (0.86–2.70)	0.150	38.4	1.25 (0.93–1.69)	0.139	
Completion of university degree (n=392)	35.7	1.00		5.6	1.00		36.7	1.00		
Income personal	
Low income (0–199%) (n=439)	43.1	1.42 (0.97–2.06)	0.069	10.3	3.92 (1.52–10.07)	0.005	40.5	1.40 (0.96–2.04)	0.080	
Middle income 
(200–399%) (n=371)	34.8	0.94 (0.64–1.38)	0.762	6.2	2.20 (0.82–5.89)	0.118	36.1	1.09 (0.74–1.61)	0.650	
High income >=400% (n=167)	37.1	1.00		3.0	1.00		36.5	1.00		
Income family	
Woman does not know (n=323)	37.8	1.83 (1.06–3.14)	0.030	7.4	6.67 (0.89–50.10)	0.065	36.2	1.75 (1.01–3.01)	0.044	
Low income (0–199%) (n=283)	48.1	2.31(1.34–4.00)	0.003	14.1	13.23 (1.78–98.09)	0.012	43.1	2.00 (1.16–3.47)	0.013	
Middle income 
(200–399%) (n=233)	41.6	1.84 (1.05–3.20)	0.033	3.9	3.25 (0.41–26.11)	0.267	40.3	1.79 (1.02–3.13)	0.041	
High income >=400% (n=85)	25.9	1.00		1.2	1.00		27.1	1.00		
