# Supplementary material for: Inequalities in health and health service utilisation among reproductive age women in St. Petersburg, Russia: a cross-sectional study
Source: BMC Health Serv Res. 2010 Nov 11;10:307. doi: 10.1186/1472-6963-10-307 (PMC2992514; doi:10.1186/1472-6963-10-307)
Supplement: Additional file 3 — Table S3 "Prevalence and age - adjusted OR (95% CI) for use of different health care providers by SES" is included into the file. [file 1472-6963-10-307-S3.RTF]

Table 3. Prevalence and age - adjusted OR (95% CI) for use of different health care providers by SES.

Socioeconomic characteristics	Policlinic	Private	Occupational	Hospital admission	
	%	OR (95% CI)	p-value	%	OR (95% CI)	p-value	%	OR (95% CI)	p-value	%	OR (95% CI)	p-value	
Education		
School or college (n=339)	40.4	1.00		5.6	1.00		2.7	1.00		17.9	1.00		
Some university studies (n=409)	40.6	0.98 (0.73–1.32)	0.876	9.8	1.73 (0.97–3.06)	0.062	5.1	2.28 (1.02–5.09)	0.044	13.8	0.69 (0.46–1.03)	0.071	
Completion of university degree (n=392)	41.6	1.05 (0.78–1.42)	0.734	16.6	3.37(1.98–5.76)	<0.001	6.6	2.60 (1.20–5.63)	0.016	13.1	0.69 (0.46–1.04)	0.078	
Personal income	
Low income (0–199%) (n=439)	41.5	1.00		8.4	1.00		3.4	1.00		15.9	1.00		
Middle income 
(200–399%) (n=371)	45.8	1.21 (0.91–1.60)	0.189	7.8	0.94 (0.57–1.57)	0.823	6.2	1.83 (0.94–3.56)	0.077	14.3	0.89 (0. 60–1.31)	0.550	
High income >=400% 
(n=167)	36.5	0.84 (0.58–1.21)	0.351	23.4	3.56 (2.16–5.86)	<0.001	5.4	1.54 (0.66–3.59)	0.321	12.0	0.75 (0.44–1.27)	0.283	
Family income	
Low income (0–199%) (n=283)	46.3	1.00		7.4	1.00		4.6	1.00		16.2	1.00		
Middle income 
(200–399%) (n=233)	40.3	0.78 (0.55–1.10)	0.156	12.9	1.81 (1.01–3.27)	0.048	6.9	1.61 (0.76–3.43)	0.218	15.5	0.93 (0.57–1.50)	0.754	
High income >=400% 
(n=85)	41.2	0.80 (0.49 –1.32)	0.386	18.8	2.81 (1.38–5.71)	0.004	4.7	1.21 (0.38–3.85)	0.751	14.3	0.82 (0.41–1.64)	0.572	
Woman does not know (n=323)	38.7	0.70 (0.50–0.98)	0.038	11.8	1.56 (0.87–2.78)	0.135	4.3	1.16 (0.52–2.59)	0.710	15.7	0.87 (0.55–1.38)	0.550	
