# Supplementary material for: Inequalities in health and health service utilisation among reproductive age women in St. Petersburg, Russia: a cross-sectional study
Source: BMC Health Serv Res. 2010 Nov 11;10:307. doi: 10.1186/1472-6963-10-307 (PMC2992514; doi:10.1186/1472-6963-10-307)
Supplement: Additional file 4 — Table S4 "Prevalence and age-adjusted OR for different types of health practices use during pregnancy by SES" is included into the file. [file 1472-6963-10-307-S4.RTF]

Table 4. Prevalence and age-adjusted OR for different types of health practices use during pregnancy by SES.

Socioeconomic characteristic	Women clinic	Policlinic
	Private	Other centres	
	%	OR (95% CI)	p-value	%	OR (95% CI)	p-value	%	OR (95% CI)	p-value	%	OR (95% CI)	p-value	
Education		
School or college
(n=126)	95.0	1.00		19.3	1.00		2.5	1.00		6.7	1.00		
Some university studies (n=99)	86.9	0.37 (0.13–1.00)	0.050	12.1	0.62 (0.29–1.32)	0.213	13.1	6.40 (1.75–23.34)	0.005	13.1	2.16 (0.85–5.47)	0.104	
Completion of university degree
(n=119)	96.8	1.70 (0.47–6.20)	0.422	19.0	1.10 (0.57–2.10)	0.783	11.9	5.90 (1.64–21.20)	0.007	14.3	2.41 (1.00–5.81)	0.051	
Personal income		
Low income (0–199%) (n=155)	92.9	1.00		18.7	1.00		5.8	1.00		9.7	1.00		
Middle income 
(200–399%) (n=89)	94.4	1.30 (0.44–3.89)	0.635	18.0	1.00 (0.50–1.98)	0.998	10.1	2.02 (0.76–5.37)	0.161	10.1	1.09 (0.45–2.62)	0.845	
High income >=400% 
(n=47)	91.5	0.82 (0.25–2.72)	0.746	10.6	0.45 (0.15–1.35)	0.153	6.4	1.32 (0.33–5.19)	0.694	14.9	1.79 (0.68–4.76)	0.240	
Family income		
Low income (0–199%) (n=116)	93.1	1.00		22.4	1.00		6.0	1.00		8.6	1.00		
Middle income 
(200–399%) (n=67)	91.0	0.74 (0.25–2.25)	0.599	14.9	0.60 (0.27–1.34)	0.213	13.4	2.46 (0.86–7.00)	0.092	10.4	1.23 (0.45–3.41)	0.689	
High income >=400% 
(n=20)	90.0	0.63 (0.12–3.23)	0.581	10.0	0.20 (0.03–1.60)	0.130	5.0	0.96 (0.11–8.35)	0.969	15.0	2.05 (0.51–8.31)	0.313	
Woman does not know 
(n=94)	94.7	1.25 (0.39–4.01)	0.702	13.8	0.48 (0.23–1.02)	0.057	9.6	1.45 (0.51–4.10)	0.486	14.9	1.74 (0.73–4.14)	0.214	
