# Supplementary material for: Inequalities in health and health service utilisation among reproductive age women in St. Petersburg, Russia: a cross-sectional study
Source: BMC Health Serv Res. 2010 Nov 11;10:307. doi: 10.1186/1472-6963-10-307 (PMC2992514; doi:10.1186/1472-6963-10-307)
Supplement: Additional file 5 — Table S5 "Prevalence and age-adjusted OR for preventive health care examinations by education and income" is included into the file. [file 1472-6963-10-307-S5.RTF]

Table 5. Prevalence and age-adjusted OR for preventive health care examinations by education and income.
Socioeconomic 
characteristic	Mammography
under 40
(n=801)	Mammography
40+
(n=344)	Ultrasonic examination of breast
(n=1147)	PAP smear
(n=1103)	Gynaecological examination
(n=1147)	
	%	OR
(95% CI)	p-value	%	OR
(95% CI)	p-value	%	OR
(95% CI)	p-value	%	OR
(95% CI)	p-value	%	OR
(95% CI)	p-value	
Education		
School or college
(n=223a/116b/339c/331d)	4.5	1.00		11.2	1.00		9.1	1.00		29.9	1.00		61.7	1.00		
Some university studies
(n=313/96/409/383)	6.7	1.92
(0.87–4.21)	0.104	13.5	1.24
(0.55–2.82)	0.607	11.2	1.40
(0.86–2.28)	0.177	35	1.33
(0.97–1.83)	0.082	63.3	1.05
(0.78–1.42)	0.753	
Completion of university degree
(n=262/130/392/382)	7.6	1.69
(0.77–3.72)	0.189	20.0	1.98
(0.97–4.07)	0.063	12.8	1.45
(0.90–2.32)	0.129	46.9	2.06
(1.51–2.81)	<0.001	72.2	1.62
(1.19–2.21)	0.002	
Personal income		
Low income (0–199%) (n=313/126/439/410)	4.5	1.00		11.9	1.00		9.1	1.00		33.7	1.00		62.4	1.00		
Middle income 
(200–399%) (n=244/127/371/365)	4.5	1.00
(0.44–2.25)	0.996	14.2	1.23
(0.59–2.57)	0.584	11.9	1.31
(0.83–2.07)	0.241	37.3	1.16
(0.87–1.56)	0.313	68.5	1.32
(0.98–1.77)	0.064	
High income >=400% 
(n=111/55/167/167)	10.8	2.22
(0.98–5.00)	0.055	30.9	3.32
(1.51–7.30)	0.003	18.0	2.09
(1.25–3.50)	0.005	46.7	1.72
(1.19–2.48)	0.004	71.3	1.56
(1.05–2.30)	0.027	
Family income	
Low income (0–199%) (n=166/117/283/274)	5.4	1.00		10.3	1.00		10.2	1.00		39.1	1.00		67.5	1.00		
Middle income 
(200–399%) (n=141/92/233/229)	7.1	1.52
(0.59–3.90)	0.382	13.0	1.31
(0.56–3.06)	0.539	10.3	1.04
(0.59–1.85)	0.883	43.7	1.24
(0.87–1.77)	0.240	71.7	1.20
(0.82–1.76)	0.341	
High income >=400% 
(n=66/18/85/85)
	6.1	1.30
(0.38–4.43)	0.675	33.3	4.34
(1.38–3.70)	0.012	14.1	1.64
(0.79–3.41)	0.187	38.8	1.08
(0.65–1.79)	0.766	69.4	1.09
(0.64–1.87)	0.744	
Woman does not know 
(n=265/58/323/229)	4.2	0.97
(0.38–2.46)	0.946	19.0	2.05
(0.84–4.97)	0.114	9.3	1.05
(0.60–1.85)	0.854	32.4	0.82
(0.57–1.17)	0.265	59.8	0.68
(0.48–0.96)	0.028	
a n for women under 40 years of age
b n for women of 40+
c n for the whole sample
d n for Pap smear analysis, those who never had sexual intercourse are excluded
